# Antibiotics Improve the Treatment Efficacy of Oxaliplatin-Based but Not Irinotecan-Based Therapy in Advanced Colorectal Cancer Patients

**DOI:** 10.1155/2020/1701326

**Published:** 2020-06-17

**Authors:** Hiroo Imai, Ken Saijo, Keigo Komine, Yuya Yoshida, Keiju Sasaki, Asako Suzuki, Kota Ouchi, Masahiro Takahashi, Shin Takahashi, Hidekazu Shirota, Masanobu Takahashi, Chikashi Ishioka

**Affiliations:** Department of Medical Oncology, Tohoku University Hospital, 4-1, Seiryo-machi, Aobaku, Sendai 980-8575, Japan

## Abstract

**Background:**

Oxaliplatin and irinotecan are generally used to treat advanced colorectal cancer (CRC) patients. Antibiotics improve the cytotoxicity of oxaliplatin but not irinotecan in a colon cancer cell line *in vitro*. This study retrospectively assessed whether antibiotics improve the treatment efficacy of oxaliplatin- but not irinotecan-based therapy in advanced CRC patients. *Patients and Methods*. The medical records of 220 advanced CRC patients who underwent oxaliplatin- or irinotecan-based therapy were retrospectively reviewed. The oxaliplatin and irinotecan groups were further divided into antibiotic-treated (group 1) and antibiotic-untreated (group 2) subgroups.

**Results:**

In oxaliplatin groups 1 and 2, the response rate (RR) was 58.2% and 30.2%, while the disease control rate (DCR) was 92.5% and 64.2%, respectively; the median progression-free survival (PFS) was 10.5 months (95% confidence interval (CI) = 7.5–12.2) and 7.0 months (95% CI = 17.0–26.0), respectively, and the median overall survival (OS) was 23.8 months (95% CI = 5.1–9.1) and 17.4 months (95% CI = 13.1–24.9), respectively. In irinotecan groups 1 and 2, the RR was 17.8% and 20.0%, while the DCR was 75.6% and 69.1%, respectively; the median PFS was 8.2 months (95% CI = 6.2–12.7) and 7.9 months (95% CI = 12.0–23.0), respectively, and the median OS was 16.8 months (95% CI = 5.9–10.6) and 13.1 months (95% CI = 10.4–23.7), respectively.

**Conclusion:**

To improve the treatment efficacy of oxaliplatin-based therapy in advanced CRC patients, adding antibiotics is a potential therapeutic option.

## 1. Introduction

Oxaliplatin and irinotecan are anticancer agents used to treat cancer patients [1, 2] and in colorectal cancer (CRC) patients, oxaliplatin-, and irinotecan-based therapy is generally used as first- or second-line treatment [3–6]. However, their treatment efficacy in advanced CRC patients is limited. Some types of bacteria mediate the resistance to gemcitabine in the pancreatic cancer cell line and to oxaliplatin but not to irinotecan in the colon cancer cell line [7]. A retrospective study revealed that antibiotics improve the treatment efficacy of gemcitabine-based therapy in advanced cancer patients [8]. We hypothesized that improvement in the treatment efficacy of cytotoxic anticancer agents by adding antibiotics is independent of the tumor primary site but dependent on the type of anticancer agent. This retrospective study assessed whether antibiotics improve the treatment efficacy of oxaliplatin-based but not irinotecan-based therapy in advanced CRC patients.

## 2. Methods

### 2.1. Patients

The medical records (2011–2018) of patients diagnosed with CRC histopathologically and administered oxaliplatin- or irinotecan-based therapy as first- or second-line treatment were retrospectively reviewed at the Department of Medical Oncology, Tohoku University Hospital, Japan. Inclusion criteria were as follows: (1) patients with histologically confirmed colorectal adenocarcinoma, (2) patients with least one measurable cancer lesion, (3) patients with unresectable or metastatic lesions, (4) patients who underwent at least one course of oxaliplatin- or irinotecan-based therapy, and (5) patients in whom the treatment efficacy of oxaliplatin-or irinotecan-based therapy had been assessed by computed tomography (CT) at least once. Patients who did not met these inclusion criteria were excluded from the study.

Finally, we identified 120 patients who underwent oxaliplatin-based therapy and 100 patients who underwent irinotecan-based therapy. The oxaliplatin group was further subdivided into antibiotic-treated (group 1; *n* = 67) and antibiotic-untreated (group 2; *n* = 53) groups. Similarly, the irinotecan group was further subdivided into antibiotic-treated (group 1; *n* = 53) and antibiotic-untreated (group 2; *n* = 47) groups.

The study protocol was approved by the ethics committee of Tohoku University Hospital.

### 2.2. Treatment Methods

The oxaliplatin-based therapy procedures in this study were as follows:

- mFOLFOX6: 85 mg/m^2^ of oxaliplatin and 200 mg/m^2^ of leucovorin given intravenously over 2 h, followed immediately by 400 mg/mg^2^ of a fluorouracil (5-FU) intravenous bolus and then 2400 mg/m^2^ of 5-FU as a 46 h infusion
- SOX: 80 mg/m^2^ of S-1 orally administered on days 1–14 and 130 mg/m^2^ of oxaliplatin given intravenously on day 1
- CapeOX: 1000 mg/^2^ of capecitabine orally administered twice a day on days 1–14 and 130 mg/m^2^ of oxaliplatin given intravenously on day 1

The irinotecan-based therapy procedures were as follows:

- mFOLFIRI: 150 mg/m^2^ of irinotecan and 200 mg/m^2^ of leucovorin given intravenously over 2 h, followed immediately by 400 mg/mg^2^ of a 5-FU intravenous bolus and then 2400 mg/m^2^ of 5-FU given as a 46 h infusion
- S-1 plus irinotecan: 80 mg/m^2^ of S-1 orally administered on days 1–14 and 125 mg/m^2^ of irinotecan given intravenously on day 1
- Irinotecan alone: 125 mg/m^2^ of irinotecan given twice a week intravenously

Both oxaliplatin group 1 and irinotecan group 1 were administered antibiotics from 2 weeks before the start of oxaliplatin- or irinotecan-based therapy, respectively, to the first imaging evaluation of the treatment efficacy of oxaliplatin- or irinotecan-based therapy, respectively, using CT.

### 2.3. Evaluation

Responses were assessed using the *Response Evaluation Criteria in Solid Tumors* version 1.0 [9]. The complete response (CR; all signs of cancer disappeared after oxaliplatin- or irinotecan-based therapy) and partial response (PR; ≥30% decrease in the diameter of measurable lesions on CT) rates were combined and defined as the response rate (RR). The CR, PR, and stable disease (SD; <30% decrease and <20% increase in the diameter of measurable lesions on CT) rates were combined and defined as the disease control rates (DCR). The relative dose intensity of oxaliplatin- or irinotecan was defined as the ratio of the total actual dose to the planned dose. Hematological toxicity was reviewed from medical records and evaluated according to the Common Terminology Criteria for Adverse Events version 4.0 [10].

### 2.4. Statistical Analysis

The median progression-free survival (PFS) and the median overall survival (OS) were calculated using the Kaplan–Meier method. *P* values of the RR and DCR between groups 1 and 2 were based on Fisher's exact test. Univariate and multivariate analyses were performed for the relationship between the response to oxaliplatin- or irinotecan-based therapy and the patients' background and severe neutropenia. Statistical analyses, including univariate analysis, multivariate analysis, Pearson's chi-square test, and Fisher's exact test, were performed using JMP® 11 (SAS Institute Inc., Cary, NC, USA). *P* < 0.05 was considered statistically significant.

## 3. Results

### 3.1. Patient Characteristics

Patient characteristics are presented in Table 1. The number of patients who underwent oxaliplatin-based therapy as first- or second-line treatment was comparable between groups 1 and 2. Similarly, the number of patients who underwent irinotecan-based therapy as first- or second-line treatment was comparable between groups 1 and 2. The number of patients who underwent surgery for their primary lesion was significantly high in oxaliplatin group 2 compared to group 1 (Table 1). However, univariate and multivariate analysis showed that the imbalance in the ratio of patients who underwent surgery between oxaliplatin groups 1 and 2 does not influence the correlation between antibiotic treatment and the treatment efficacy of oxaliplatin-based therapy.

### 3.2. Efficacy of Oxaliplatin- or Irinotecan-Based Therapy

The RR and DCR of both oxaliplatin and irinotecan groups 1 and 2 are given in Table 2. Both RR and DCR were significantly high in oxaliplatin group 1 compared to oxaliplatin group 2. The RR of oxaliplatin groups 1 and 2 was 58.2% and 30.2%, respectively, while the DCR was 92.5% and 64.2%, respectively. In contrast, there was no significant difference in the RR and DCR between irinotecan groups 1 and 2. The RR of irinotecan groups 1 and 2 was 17.8% and 20.0%, respectively, while the DCR was 75.6% and 69.1%, respectively.

**Figure 1:**
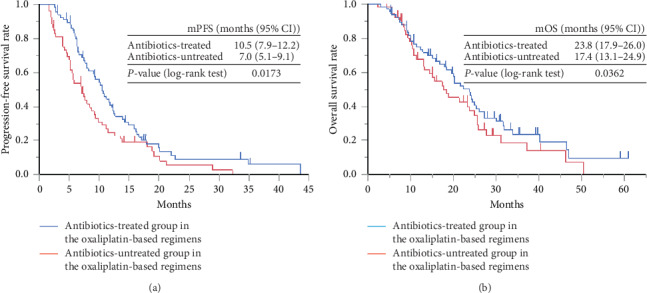
Kaplan–Meier curve of the (a) PFS and (b) OS of antibiotic-treated group (group 1) and antibiotic-untreated group (group 2) in oxaliplatin-based therapy. PFS: progression-free survival; OS: overall survival.

Both median PFS and median OS were significantly long in oxaliplatin group 1 compared to oxaliplatin group 2. As shown in Figure 1(a), the median PFS of oxaliplatin groups 1 and 2 was 10.5 months (95% confidence interval [CI] = 7.5–12.2) and 7.0 months (95% CI = 5.1–9.1), respectively. As shown in Figure 1(b), the median OS of oxaliplatin groups 1 and 2 was 23.8 months (95% CI = 17.9–26.0) and 17.4 months (95% CI = 13.1–24.9), respectively.

**Figure 2:**
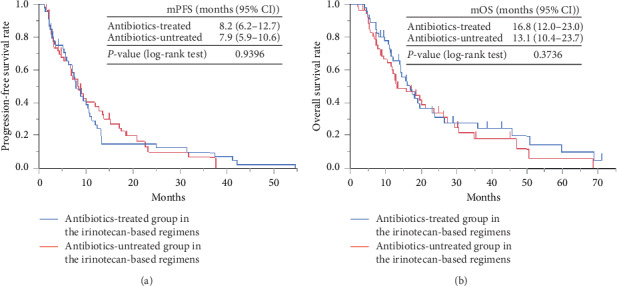
Kaplan–Meier curve of the (a) PFS and (b) OS of the antibiotic-treated group (group 1) and antibiotic-untreated group (group 2) in irinotecan-based therapy. PFS: progression-free survival; OS: overall survival.

In contrast, there was no significant difference in the median PFS and median OS between irinotecan groups 1 and 2. As shown in Figure 2(a), the median PFS of irinotecan groups 1 and 2 was 8.2 months (95%CI = 6.2–12.7) and 7.9 months (95% CI = 5.9–10.6), respectively. As shown in Figure 2(b), the median OS of irinotecan groups 1 and 2 was 16.8 months (95% CI = 12.0–23.0) and 13.1 months (95% CI = 10.4–23.7), respectively.

We divided oxaliplatin groups 1 and 2 into two groups, respectively. In each group, patients who were treated with fluoropyrimidine intravenously were assigned to oxaliplatin-1-mFOLFOX6 group and oxaliplatin-2-mFOLFOX6 group. In each group, patients who were treated with fluoropyrimidine orally were assigned to oxaliplatin-1-SOX/CapeOX group and oxaliplatin-2-SOX/CapeOX group. We compared the response rate, median PFS and the median OS between oxaliplatin-1-mFOLFOX6 group and oxaliplatin-1-SOX/CapeOX group or between oxaliplatin-2-mFOLFOX6 group oxaliplatin-2-SOX/CapeOX group, respectively. As shown in Supplemental Figure 1 and Supplemental Figure 2, there was no significant difference in median PFS or median OS between oxaliplatin-1-mFOLFOX6 and oxaliplatin-1-SOX/CapeOX or between oxaliplatin-2-mFOLFOX6 group and oxaliplatin-2-SOX/CapeOX group, respectively.

As described in Supplemental Table 1, there was no significant difference in response rate between oxaliplatin-1-mFOLFOX6 and oxaliplatin-1-SOX/CapeOX or between oxaliplatin-2-mFOLFOX6 group and oxaliplatin-2-SOX/CapeOX group, respectively.

### 3.3. Hematological Toxicity

Hematological toxicity values of both oxaliplatin and irinotecan groups 1 and 2 are given in Table 3. The number of patients with severe leukopenia and neutropenia in oxaliplatin group 1 was significantly high compared to oxaliplatin group 2. The anemia and thrombocytopenia incidence rates and the increase in bilirubin, liver transaminase, and creatinine were similar in oxaliplatin groups 1 and 2. Similarly, the number of patients with severe leukopenia and neutropenia in irinotecan group 1 was significantly high compared to irinotecan group 2. The leukopenia, anemia, and thrombocytopenia incidence rates and the increase in bilirubin, transaminase, and creatinine were similar in irinotecan groups 1 and 2.

### 3.4. Univariate and Multivariate Analyses

Results of univariate and multivariate analyses are shown in Table 4. We found a statistically significant relationship between the response to oxaliplatin-based therapy and antibiotic treatment (univariate analysis: *P*=0.0159; multivariate analysis: *P*=0.0114). The other seven factors were not significantly correlated with the response to oxaliplatin-based therapy. In addition, all eight factors were not significantly correlated with the response to irinotecan-based therapy.

## 4. Discussion

A previous study [7] revealed that a decrease in intratumor bacteria by antibiotic treatment augments the antitumor efficacy of gemcitabine in tumor-bearing mice. On the basis of that report [7], we retrospectively demonstrated that antibiotic treatment augments the treatment efficacy of gemcitabine-based therapy in advanced cancer patients [8]. In addition, a decrease in bacteria by adding antibiotics also augments the cytotoxicity of oxaliplatin but not of irinotecan in the CRC cell line *in vitro* [7], which is consistent with our results in this study. Antibiotic treatment is a factor that is significantly correlated with the efficacy of oxaliplatin-based therapy in advanced CRC patients.

Patients with leukopenia or neutropenia are generally administered antibiotics for prophylaxis [11]. Therefore, it is inevitable that a high number of advanced CRC patients administered antibiotics get leukopenia or neutropenia compared to advanced CRC patients not administered antibiotics. This seems to indicate that adding antibiotics to oxaliplatin- or irinotecan-based therapy does not increase cytotoxicity by oxaliplatin- or irinotecan-based therapy in advanced CRC patients. In addition, there seems to be no correlation between an increase in the incidence rate of leukopenia and improvement in the treatment efficacy of anticancer agents. However, it has been reported that 5-FU and oxaliplatin combination therapy for patients with advanced colorectal cancer has stronger myelotoxicity than SOX therapy or CapeOX therapy for patients with advanced colorectal cancer [12, 13]. There was a higher rate of patients who were treated with mFOLFOX6 regimen in oxaliplatin group 1 when compared to those in oxaliplatin group 2. This might be one explanation to the reason why there were a higher rate of leukopenia and neutropenia in oxaliplatin group 1 than in oxaliplatin group 2.

It has been reported that the primary resection of colorectal cancer worsens the prognosis of patients with advanced colorectal cancer [14]. There was a significantly higher resection rate of the primary site in oxaliplatin group 2 when compared to those in oxaliplatin group 1. The shorter overall survival time of oxaliplatin group 2 when compared to that of oxaliplatin group 1 might be partly attributable to the higher rate of the resection of the primary site in oxaliplatin group 2.

A previous study reported improvement in the treatment efficacy of anticancer agents by adding antibiotics in pancreatic cancer patients [8], indicating that this improvement is independent of the tumor primary site. In contrast, improvement in the treatment efficacy of anticancer agents by adding antibiotics seems to depend on the type of anticancer agent.

This study had a few limitations. First, the study had a retrospective design. Second, the number of patients included was relatively small. Third, cancer type studies were limited to CRC. Both gastric cancer and pancreatic cancer patients undergo oxaliplatin-based therapy in clinical practice [15, 16]. In addition, lung cancer, gastric cancer, and ovarian cancer patients undergo irinotecan-based therapy [2, 17, 18]. However, we did not assess the treatment efficacy and safety of adding antibiotics to oxaliplatin- or irinotecan-based therapy in patients with these types of cancer. Fourth, we could not obtain data on the incidence rate of nonhematological toxicities such as diarrhea. There are trillions of bacteria in the intestinal mucosa [19]. Antibiotic treatment decreases the number of bacteria in the intestinal mucosa and should augment the cytotoxicity of oxaliplatin in the intestinal mucosa, thereby increasing diarrhea. Fifth, we did not assess whether bacteria exist in tumor tissue pathologically in our patients. *Fusobacterium nucleatum*, which is part of the gut microbiome, is strongly associated with the tumorigenesis of CRC [20] and infiltrates cancer tissue in advanced CRC patients [21, 22]. It is possible that in a large proportion of our patients, bacteria infiltrated CRC tissue. Therefore, improvement in the treatment efficacy of oxaliplatin-based therapy might be attributable to a decrease in bacteria in tumor tissue by adding antibiotics. Further prospective or retrospective studies are required in order to overcome these limitations.

## 5. Conclusion

Adding antibiotics is a potential therapeutic option to improve the treatment efficacy of oxaliplatin-based but not irinotecan-based therapy in advanced CRC patients. Prospective or retrospective studies to assess the treatment efficacy and safety of adding antibiotics to oxaliplatin- or irinotecan-based therapy are warranted.

## Figures and Tables

**Table 1:** Background of patients who underwent oxaliplatin- or irinotecan-based therapy (*n* = 220).

Group	Oxaliplatin		*P*	Irinotecan		*P*
	Antibiotic-treated (*n* = 67)	Antibiotic-untreated (*n* = 53)		Antibiotic-untreated (*n* = 53)	Antibiotic-untreated (*n* = 47)	
Number	67	53		53	47	
Sex						
Male	38 (56.7)	29 (54.7)	0.9901	33 (62.2)	26 (55.3)	0.481
Female	29 (43.3)	24 (45.3)		20 (37.8)	21 (44.7)	
Age (mean)	68 (27–86)	66 (24–83)		70 (32–80)	66 (38–83)	
Kras status						
Wild type	35 (52.2)	28 (52.8)	0.6653	22 (41.5)	26 (55.3)	0.1677
Mutant type	32 (48.8)	25 (47.2)		31 (58.5)	21 (44.7)	
Line of oxaliplatin-based or irinotecan-based chemotherapy						
First line	45 (67.1)	36 (67.9)	0.7569	21 (39.6)	15 (31.9)	0.4229
Second-line	22 (32.8)	17 (32.1)		32 (60.4)	32 (68.1)	
Primary site						
Left side colon	48 (71.6)	30 (56.6)	0.1314	31 (58.5)	33 (70.2)	0.2229
Right side colon	19 (28.4)	23 (43.4)		22 (41.5)	14 (29.8)	
Relative dose intensity of oxaliplatin (%)	72.1	71.3		69.4	65.8	
Resection of primary site						
(+)	39 (58.2)	47 (88.7)	0.002	37 (69.8)	36 (76.6)	0.4456
(−)	28 (41.8)	6 (11.3)		16 (30.2)	11 (23.4)	
Regimen of first-line chemotherapy (%)						
FOLFOX (plus bmab or cmab or pmab)	53 (79.1)	33 (62.3)	0.0521			
SOX (plus bmab)	9 (13.4)	12 (22.6)	0.1874			
CapeOX (plus bmab)	5 (7.5)	8 (16.9)	0.1816			
FOLFORI (plus bmab or rmab or AFL or cmab or pmab)				38 (71.7)	32 (68.1)	0.6939
S-1 plus irinotecan				13 (24.5)	13 (27.7)	0.7216
CPT11				2 (3.8)	2 (4.3)	0.9023
Average number of treatment (range)						
FOLFOX (plus bmab or cmab or pmab)	17.1 (4–28)	18.9 (6–29)				
SOX (plus bmab)	10.5 (4–14)	11.2 (5–13)				
CapeOX (plus bmab)	9.5 (3–12)	10.1 (4–13)				
FOLFORI (plus bmab or rmab or AFL or cmab or pmab)				16.9 (5–22)	15.8 (4–23)	
S-1 plus irinotecan				10.5 (4–16)	11.8 (5–15)	
CPT11				16.2 (5–18)	15.8 (4–21)	
Postchemotherapy						
Irinotecan-based chemotherapy	35 (52.2)	27 (50.9)	0.8879	0 (0)	0 (0)	1
Oxaliplatin-based chemotherapy	0 (0)	0 (0)	1	21 (39.6)	15 (31.9)	0.4229
Cmab or pmab plus irinotecan	7 (10.4)	5 (9.4)	0.9011	5 (9.4)	4 (8.5)	0.8854
Trifluridine, tipiracil	19 (28.4)	15 (28.3)	0.9946	21 (39.6)	14 (29.8)	0.3034
Regorafenib	12 (17.9)	11 (20.8)	0.6943	12 (22.6)	13 (27.7)	0.563
Trastuzumab	1 (1.5)	0 (0.0)	0.3718	1 (1.9)	0 (0.0)	0.3439
Pembrolizumab	1 (1.5)	0 (0.0)	0.3718	1 (1.9)	0 (0.0)	0.3439

FOLFOX: fluorouracil (5-FU) plus oxaliplatin combination therapy; SOX: S-1 plus oxaliplatin combination therapy; CapeOX: capecitabine plus oxaliplatin combination therapy; Bmab: bevacizumab. *P* was calculated using Pearson's chi-square test.

**Table 2:** RRs and DCRs of antibiotic-treated and antibiotic-untreated groups in oxaliplatin- and irinotecan-based therapy.

Group	Oxaliplatin			Irinotecan		
	Antibiotic-treated (*n* = 67)	Antibiotic-untreated (*n* = 53)	*P*	Antibiotic-treated (*n* = 53)	Antibiotic-untreated (*n* = 47)	*P*
CR	0	0		0	0	
PR	39	19		11	8	
SD	23	21		27	26	
PD	5	13		17	11	
RR (%)	58.2	30.2	0.0224	20.8	17	0.7778
DCR (%)	92.5	64.2	0.0201	71.7	72.3	0.6724

CR: complete response; PR: partial response; SD, stable disease; PD: progressive disease; RR: response rate; DCR: disease control rate. *P* was calculated using Fisher's exact test.

**Table 3:** Hematological toxicity of antibiotic-treated and antibiotic-untreated groups in oxaliplatin- and irinotecan-based therapy.

Group	Oxaliplatin-based therapy			Irinotecan-based therapy		
	Antibiotic-treated (*n* = 67)	Antibiotic-untreated (*n* = 53)	*P*	Antibiotic-treated (*n* = 53)	Antibiotic- untreated (*n* = 47)	*P*
Leukopenia	9 (13.4)	1 (1.9)	0.042	11 (20.8)	5 (10.6)	0.156
Neutropenia	22 (32.8)	5 (9.4)	0.016	20 (37.7)	6 (12.8)	0.045
Anemia	7 (10.4)	5 (9.4)	0.854	2 (3.8)	2 (4.3)	0.751
Thrombocytopenia	3 (4.5)	5 (9.4)	0.281	5 (9.4)	4 (8.5)	0.881
Elevation of bilirubin	2 (3.0)	2 (3.8)	0.812	2 (3.8)	1 (2.1)	0.564
Elevation of AST or ALT	7 (10.4)	5 (9.4)	0.854	5 (9.4)	6 (12.8)	0.441
Elevation of creatinine	0 (0.0)	1 (1.9)	0.2	0 (0.0)	0 (0.0)	1

ALT: alanine aminotransferase; AST: aspartate aminotransferase. *P* was calculated using Pearson's chi-square test.

**Table 4:** Univariate and multivariate analyses of the relationship between the response to oxaliplatin- or irinotecan-based therapy and the patients' background and severe neutropenia.

	*n* (%)	Oxaliplatin-based therapy			Irinotecan-based therapy		
		Univariate analysis	Multivariate analysis	*P*	Univariate analysis	Multivariate analysis	*P*
		*P*	OR (95% CI)		*P*	OR (95% CI)	
Sex							
Male	67 (55.8)	0.611	1.81 (0.722–3.1222)	0.5896	0.8222	1.352 (0.745–3.089)	0.7856
Female	53 (44.2)						
Age							
≧65	68 (56.7)	0.249	1.861 (0.822–2.156)	0.3902	0.3389	1.698 (0.722–1.899)	0.4256
<65	52 (43.3)						
Antibiotics							
Untreated	53 (44.2)	0.0159	2.815 (1.656–7.228)	0.0155	0.5439	1.7989 (0.754–2.156)	0.6001
Treated	67 (55.8)						
Line of chemotherapy							
First line	81 (67.5)	0.4489	1.525 (0.758–2.115)	0.4998	0.5668	1.554 (0.564–2.225)	0.7054
Second-line	39 (32.5)						
Severe (grade 3 or 4) neutropenia							
Negative	93 (77.5)	0.5564	0.789 (0.252–2.355)	0.4655	0.6612	1.882 (0.711–2.225)	0.5154
Positive	27 (22.5)						
Operation history							
Negative	34 (28.3)	0.191	0.289 (0.896–6.283)	0.174	0.311	0.7988 (0.315–8.256)	0.3598
Positive	86 (71.7)						
Ras status							
Wild type	63 (52.5)	0.8406	1.458 (0.787–1.552)	0.7154	0.7723	1.615 (0.498–2.125)	0.782
Mutant type	57 (47.5)						
Cancer primary site							
Right side colon	42 (35.0)	0.721	0.778 (0.324–2.336)	0.747	0.6129	0.756 (0.225–3.089)	0.7255
Left side colon	78 (65.0)						

OR: odds ratio; CI: confidence interval. *P* was calculated using Pearson's chi-square test.

## Data Availability

The data used to support the findings are tables and figures included within the work. The detailed retrospective observational data used to support the findings of this study are available from the first author (Hiroo Imai, e-mail: hiroo.imai.d8@tohoku.ac.jp) upon reasonable request. All data in the current study had no personal identifiers and were kept confidential.

## Abbreviations

CRC: Colorectal cancer
OS: Overall survival
PFS: Progression-free survival
CT: Computed tomography
RR: Response rate
CR: Complete response
PR: Partial response
SD: Stable disease
DCR: Disease control rate
PD: Progressive disease
ALT: Alanine aminotransferase
AST: Aspartate aminotransferase
OR: Odds ratio
CI: Confidence interval.

## Supplementary Materials

Supplementary Materials Supplemental Table 1: RRs and DCRs of intravenous fluoropyrimidine treatment group and oral fluoropyrimidine treatment group. Supplemental Figure 1: Kaplan–Meier curve of the (a) PFS and (b) OS of oxaliplatin-1-mFOLFOX6 group and oxaliplatin-1-SOX/CapeOX group. PFS: progression-free survival; OS: overall survival. Supplemental Figure 2: Kaplan–Meier curve of the (a) PFS and (b) OS of oxaliplatin-2-mFOLFOX6 group and oxaliplatin-2-SOX/CapeOX group. PFS: progression-free survival; OS: overall survival.
