# Correction to “Antibiotics Improve the Treatment Efficacy of Oxaliplatin-Based but Not Irinotecan-Based Therapy in Advanced Colorectal Cancer Patients”

**DOI:** 10.1155/jo/9769530

**Published:** 2025-10-29

H. Imai, K. Saijo, K. Komine, et al., “Antibiotics Improve the Treatment Efficacy of Oxaliplatin-Based but Not Irinotecan-Based Therapy in Advanced Colorectal Cancer Patients,” *Journal of Oncology* 2020 (2020): 17013268, https://doi.org/10.1155/2020/1701326.

In the article titled, “Antibiotics Improve the Treatment Efficacy of Oxaliplatin-Based but Not Irinotecan-Based Therapy in Advanced Colorectal Cancer Patients,” there was an error in the abstract section. This error is shown below:

In irinotecan groups 1 and 2, the RR was 17.8% and 20.0%, while the DCR was 75.6% and 69.1%, respectively; the median PFS was 8.2 months (95% CI = 6.2–12.7) and 7.9 months (95% CI = 12.0–23.0), respectively, and the median OS was 16.8 months (95% CI = 5.9–10.6) and 13.1 months (95% CI = 10.4–23.7), respectively.

The correct text is:

In irinotecan groups 1 and 2, the RR was 17.8% and 20.0%, while the DCR was 75.6% and 69.1%, respectively; the median PFS was 8.2 months (95% CI = 6.2–12.7) and 7.9 months (95% CI = 5.9–10.6), respectively, and the median OS was 16.8 months (95% CI = 12.0–23.0) and 13.1 months (95% CI = 10.4–23.7), respectively.

We apologize for this error.

