# Supplementary material for: Antibiotics Improve the Treatment Efficacy of Oxaliplatin-Based but Not Irinotecan-Based Therapy in Advanced Colorectal Cancer Patients
Source: J Oncol. 2020 Jun 17;2020:1701326. doi: 10.1155/2020/1701326 (PMC7317329; doi:10.1155/2020/1701326)
Supplement: Supplementary Materials — Supplemental Table 1: RRs and DCRs of intravenous fluoropyrimidine treatment group and oral fluoropyrimidine treatment group. Supplemental Figure 1: Kaplan–Meier curve of the (a) PFS and (b) OS of oxaliplatin-1-mFOLFOX6 group and oxaliplatin-1-SOX/CapeOX group. PFS: progression-free survival; OS: overall survival. Supplemental Figure 2: Kaplan–Meier curve of the (a) PFS and (b) OS of oxaliplatin-2-mFOLFOX6 group and oxaliplatin-2-SOX/CapeOX group. PFS: progression-free survival; OS: overall survival. [file 1701326.f1.pdf]

Supplemental Figure 1

a)

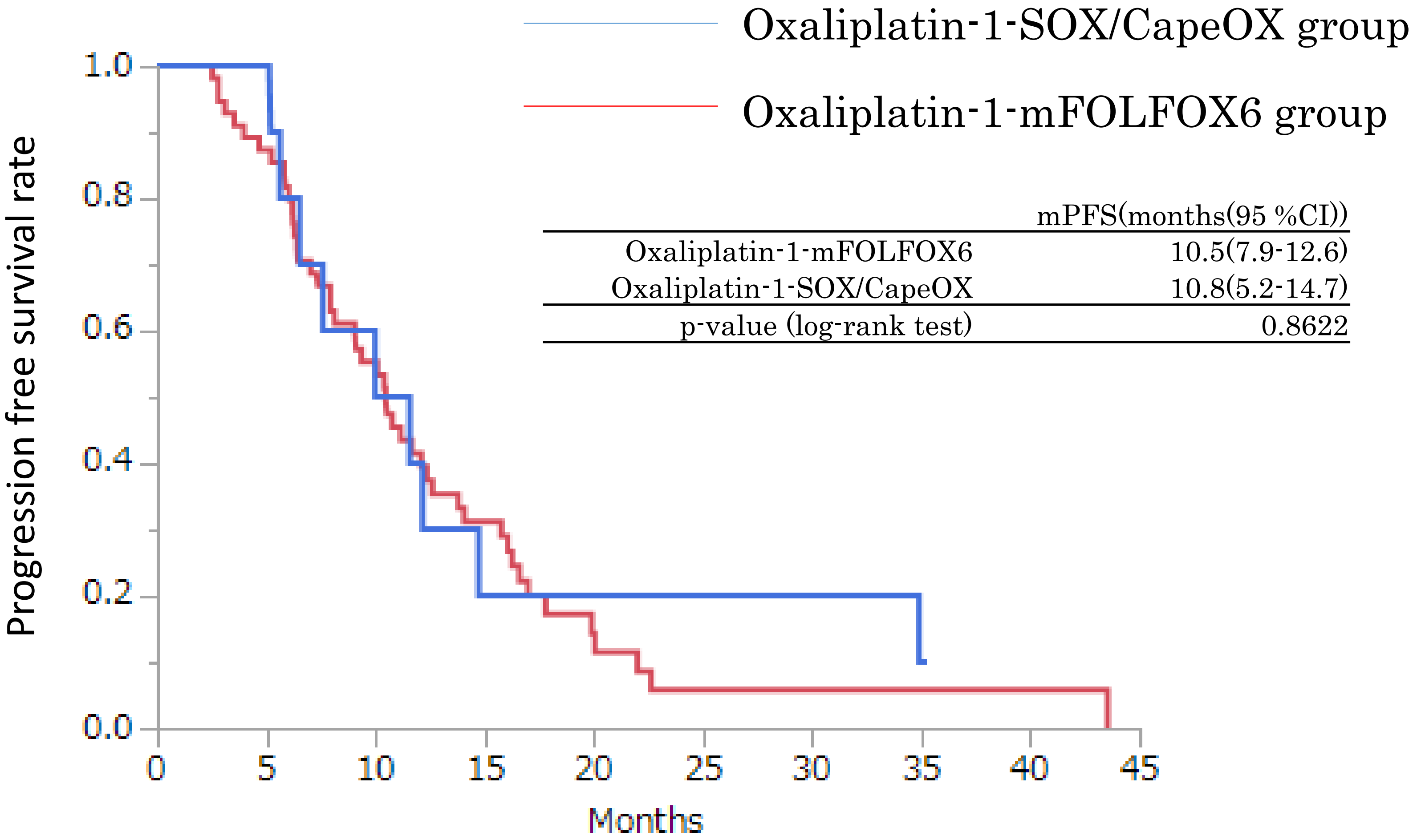

	mPFS(months(95 %CI))
Oxaliplatin-1-mFOLFOX6	10.5(7.9-12.6)
Oxaliplatin-1-SOX/CapeOX	10.8(5.2-14.7)
p-value (log-rank test)	0.8622

Supplemental Figure 1

b)

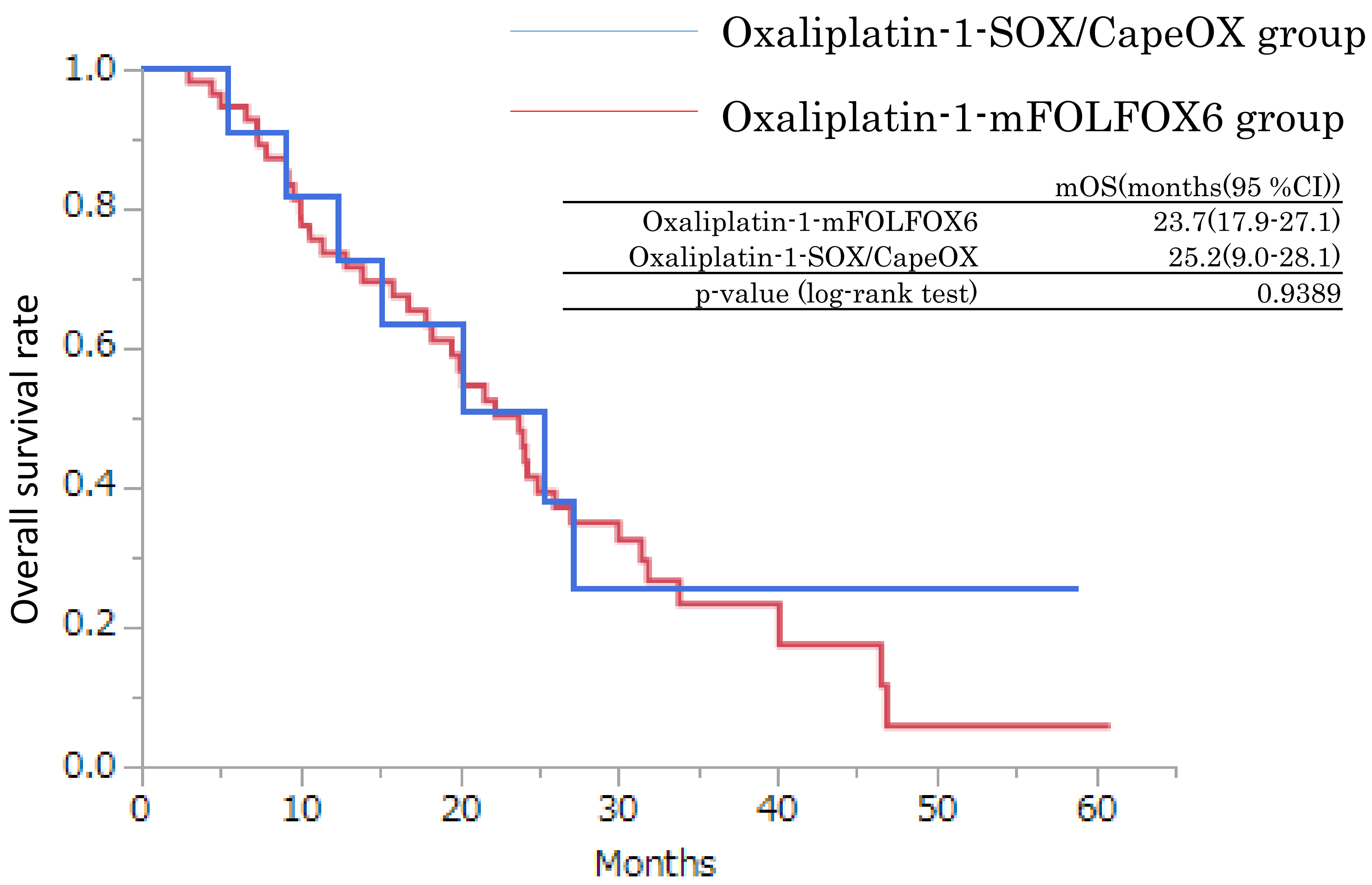

Supplemental Figure 2

a)

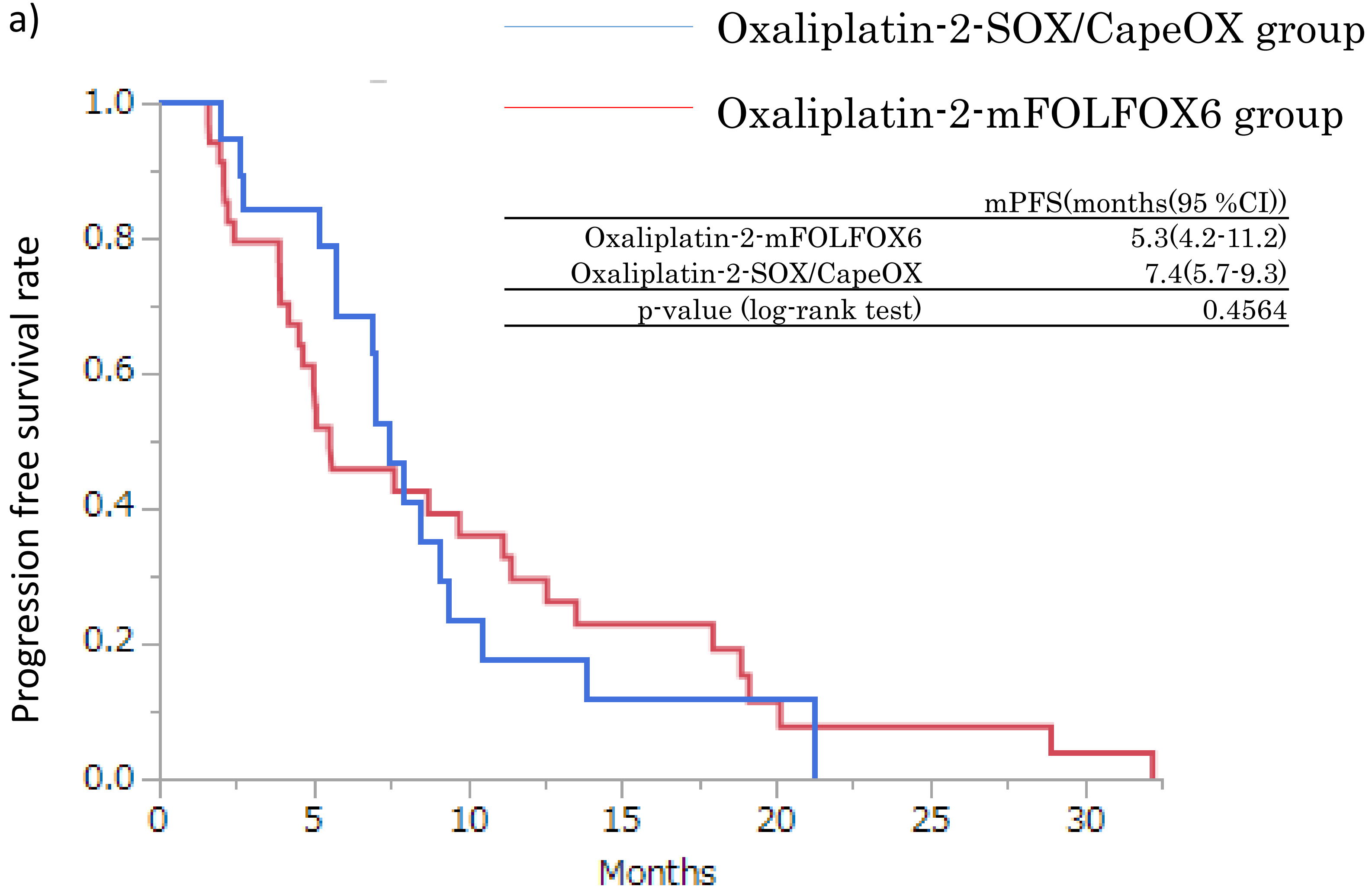

	mPFS(months(95 %CI))
Oxaliplatin-2-mFOLFOX6	5.3(4.2-11.2)
Oxaliplatin-2-SOX/CapeOX	7.4(5.7-9.3)
p-value (log-rank test)	0.4564

Supplemental Figure 2

b)

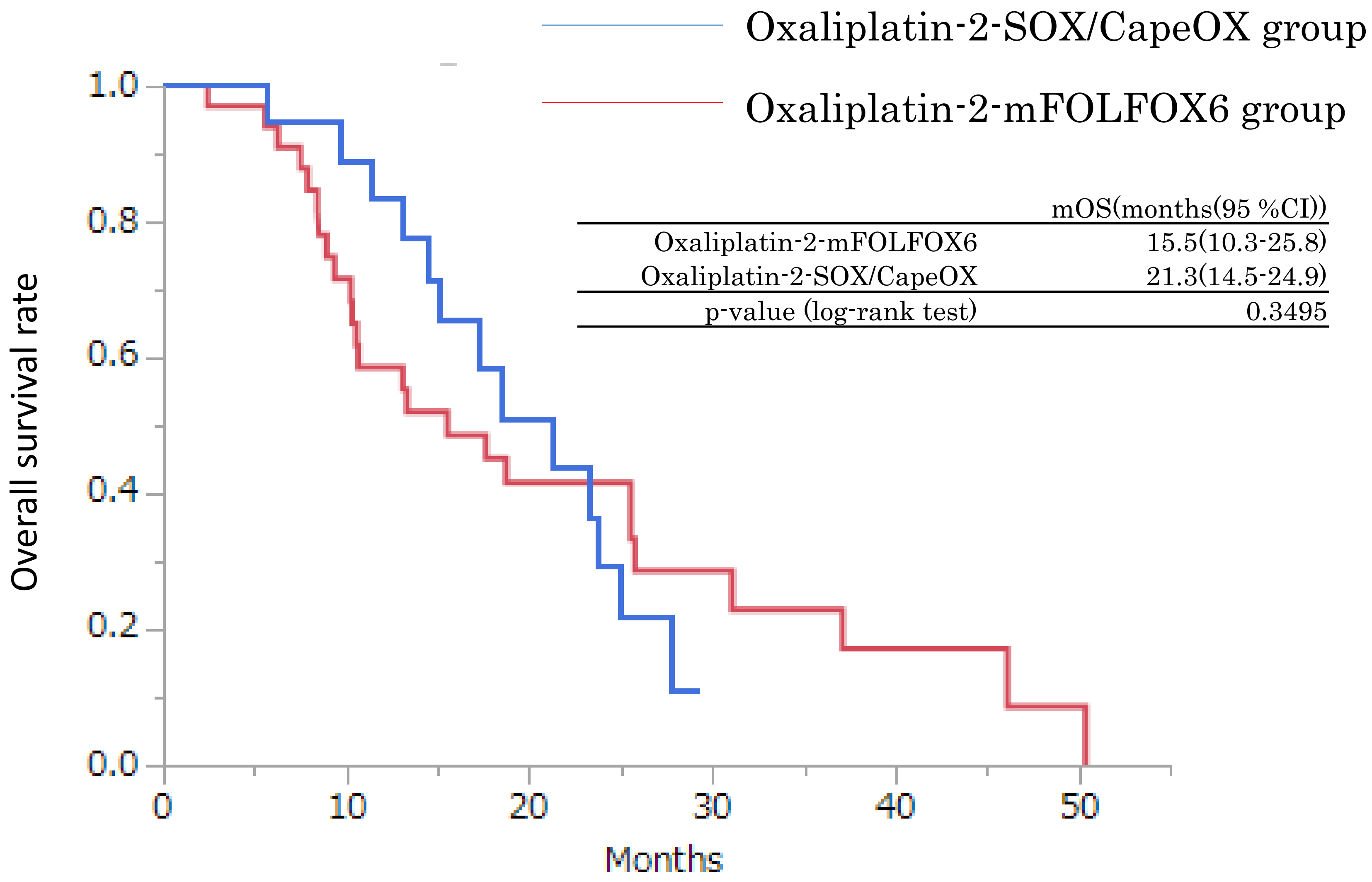

	mOS(months(95 %CI))
Oxaliplatin-2-mFOLFOX6	15.5(10.3-25.8)
Oxaliplatin-2-SOX/CapeOX	21.3(14.5-24.9)
p-value (log-rank test)	0.3495

Supplemental Table 1. RRs and DCRs of intravenous fluorropyrymidine treatment group and oral fluoropyrimidine treatment group.

Group	Oxaliplatin-1-mFOLFOX6(n=53)	Oxaliplatin-1-SOX/CapeOX(n=14)	p	Oxaliplatin-2-mFOLFOX6(n=33)	Oxaliplatin-2-SOX/CapeOX(n=20)	p
CR	0	0		0	0	
PR	30	9		11	8	
SD	20	3		13	8	
PD	3	2		9	4	
RR (%)	56.6	64.2	0.6042	33.3	40.0	0.6247
DCR (%)	94.3	85.7	0.2747	63.6	80.0	0.5508

CR, complete response; PR, partial response; SD, stable disease; PD, progressive disease; RR, response rate; DCR, disease control rate.

p was calculated using Fisher's exact test.
